# Supplementary material for: A Likelihood Approach for Real-Time Calibration of Stochastic Compartmental Epidemic Models
Source: PLoS Comput Biol. 2017 Jan 17;13(1):e1005257. doi: 10.1371/journal.pcbi.1005257 (PMC5240920; doi:10.1371/journal.pcbi.1005257)
Supplement: S1 File — (TAR.GZ) [file pcbi.1005257.s014.tar.gz › HSPH_Online-SI-Revision/PF08/n100-severe/n100-severe_PF_summary.pdf]

peak -8	R0	$1/\Theta_2$	inf	inflog	newly	newlylog	Reff	AttackRate
mean	26.5	235.3	686.5	0.6	101.6	0.3	25.6	28.5
median	26.2	238.9	261.9	0.5	39.8	0.2	25.8	25.5
Quantile .1	13.3	113.4	69.4	0.2	20.9	0.1	12.	17.4
Quantile .9	40.5	348.2	1986.8	1.2	206.6	0.4	39.3	44.3
32	0	27						
length	50							

peak -4	R0	$1/\Theta_2$	inf	inflog	newly	newlylog	Reff	AttackRate
mean	62.7	267.3	207.3	0.5	23.	0.1	56.7	50.1
median	69.2	268.7	203.	0.5	15.9	0.1	64.	48.3
Quantile .1	27.9	120.6	86.3	0.3	5.5	0.	22.9	33.6
Quantile .9	91.3	424.7	339.2	0.6	45.8	0.3	87.	69.7
0	0	0						
length	50							

peak	R0	$1/\Theta_2$	inf	inflog	newly	newlylog	Reff	AttackRate
mean	65.7	285.5	250.4	0.5	18.2	0.1	38.	55.3
median	75.9	273.6	232.5	0.5	14.7	0.1	34.	53.3
Quantile .1	29.7	120.6	66.7	0.2	2.9	0.	12.2	34.
Quantile .9	92.4	473.7	425.3	0.7	40.8	0.2	67.4	73.3
0	0	0						
length	50							

peak +4	R0	$1/\Theta_2$	inf	inflog	newly	newlylog	Reff	AttackRate
mean	64.9	282.8	1728.6	1.1	384.4	0.6	25.6	55.6
median	75.5	273.6	891.4	1.	222.6	0.5	28.7	53.1
Quantile .1	29.7	117.7	214.1	0.5	56.1	0.2	5.4	34.
Quantile .9	92.4	473.7	4576.6	1.7	950.3	1.	40.6	73.
0	0	0						
length	50							

peak +8	R0	$1/\Theta_2$	inf	inflog	newly	newlylog	Reff	AttackRate
mean	65.7	281.7	21 447.9	1.6	5019.8	1.1	43.4	56.4
median	75.9	273.6	3496.4	1.6	1019.9	1.	56.4	53.7
Quantile .1	29.7	114.	444.2	0.7	157.	0.4	14.5	34.
Quantile .9	92.4	473.7	50 297.3	2.7	11 918.8	2.1	63.5	75.1
0	0	0						
length	50							
